# Time‐resolved beam symmetry measurement for VMAT commissioning and quality assurance

**DOI:** 10.1120/jacmp.v17i2.6026

**Published:** 2016-03-08

**Authors:** Michael P. Barnes, Peter B. Greer

**Affiliations:** ^1^ Department of Radiation Oncology Calvary Mater Hospital Newcastle New South Wales Australia; ^2^ School of Medical Radiation Sciences University of Newcastle Newcastle New South Wales Australia; ^3^ School of Mathematical and Physical Sciences University of Newcastle Newcastle New South Wales Australia

**Keywords:** symmetry, VMAT, commissioning, quality assurance

## Abstract

In volumetric‐modulated arc therapy (VMAT) treatment delivery perfect beam symmetry is assumed by the planning system. This study aims to test this assumption and present a method of measuring time‐resolved beam symmetry measurement during a VMAT delivery that includes extreme variations of dose rate and gantry speed. The Sun Nuclear IC Profiler in gantry mount was used to measure time‐resolved in‐plane and cross‐plane profiles during plan delivery from which symmetry could be determined. Time‐resolved symmetry measurements were performed throughout static field exposures at cardinal gantry angles, conformal arcs with constant dose rate and gantry speed, and during a VMAT test plan with gantry speed and dose rate modulation. Measurements were performed for both clockwise and counterclockwise gantry rotation and across four Varian 21iX linacs. The symmetry was found to be generally constant throughout the static field exposures to within 0.3% with an exception on one linac of up to 0.7%. Agreement in symmetry between cardinal angles was always within 1.0% and typically within 0.6%. During conformal arcs the results for clockwise and counterclockwise rotation were in agreement to within 0.3%. Both clockwise and counterclockwise tended to vary in similar manner by up to 0.5% during arc consistent with the cardinal gantry angle static field results. During the VMAT test plan the symmetry generally was in agreement with the conformal arc results. Greater variation in symmetry was observed in the low‐dose‐rate regions by up to 1.75%. All results were within clinically acceptable levels using the tolerances of NCS Report 24 (2015).

PACS number(s): 87.55.Qr

## I. INTRODUCTION

RapidArc is the Varian version (Varian Medical Systems, Palo Alto, CA) of VMAT, which was first proposed by Otto in 2008.^(1)^ VMAT is an extension of IMRT where, as well as dynamic MLC motion and dose rate modulation, the treatment is delivered as a rotating gantry arc with potential for gantry speed modulation. The addition of gantry modulation negates the need for multiple fields and in theory the plan can be delivered with only a single arc such that a significant beam‐on time saving can be made without compromising plan quality compared to conventional IMRT. In practice, often two or more arcs are required to produce a satisfactory plan.^(2)^ The symmetry of a linear accelerator (linac) photon beam is dependent on the position and angle with which the incident electron beam strikes the target in relation to the flattening filter.

Since radiotherapy treatment planning systems (RTPS) assume a perfectly symmetric beam it is important that the beam is symmetrical for plan delivery. Before leaving the factory, the flattening filter is positioned to be centered on the collimator axis. When the beam is turned on, steering coils direct the electron beam onto the target at the correct angle and position so that the resulting photon beam impacts the flattening filter correctly. The resulting flattened beam is monitored for both positional and angular symmetry in both the transverse and radial planes by the monitor chamber, which then utilizes a servo system to feedback to the steering coils to correct the beam as necessary. The International Electrotechnical Commission (IEC) defines beam symmetry as the maximum ratio of the higher to lower absorbed dose at any two positions symmetrical to the radiation beam axis and inside the flattened area.^(3)^ The recommendation of AAPM Task Group 142^(4)^ is that, at annual QA testing, the X‐ray symmetry change from baseline be within ±1%.

While IMRT is delivered with static gantry angle, VMAT is delivered with a constantly rotating gantry with variable gantry speed and dose rate. This difference has potential implications for beam symmetry due to variable gravitational forces acting on relevant components in the linac treatment head including, but not limited to, monitor chamber, steering coils, and flattening filter. Previous studies have investigated beam symmetry for VMAT.^5^, ^6^, ^7^ Bedford and Warrington^(5)^ first looked at a method whereby symmetry was measured continuously during conformal arcs of different dose rates using the PTW LA48 linear array (PTW‐Freiburg GmbH, Freiburg, Germany). A similar test was performed by Kaurin et al.^(6)^ These tests did not examine symmetry under conditions similar to VMAT where the dose rate and gantry speed are modulated. Boylan et al.^(7)^ provided a method of testing beam stability by looking at the variation of EPID portal dosimetry pixel intensities frame by frame during a clinical VMAT delivery. By nature this method cannot provide a meaningful symmetry assessment as the beam available to be sampled is limited and varied by the MLC‐defined field at each gantry angle. It is likely that in a number of frames there will be insufficient MLC aperture size available to make a meaningful measurement in one or both directions. Also since the aperture is constantly changing during the exposure then the beam available to be sampled is constantly changing, which makes comparison at different points in the plan difficult. More recently, in 2015 the Netherlands Commission on Radiation Dosimetry (NCS) published Report 24,^(8)^ which included recommendations for VMAT linac QA. Among these recommendations were for tests that evaluated the beam symmetry stability with a fixed dose rate and static cardinal gantry angles (section 2.2.5) and during a dynamic arc treatment with dose rate and gantry speed modulation (section 2.5.1).

The aim of this work is to develop a method that can measure time‐resolved beam symmetry for VMAT under conditions similar to clinical VMAT delivery. The method is intended to meet the aims of NCS Report 24^(8)^ sections 2.2.5 and 2.5.1. The method has been applied to examine symmetry variations during VMAT compared to static gantry fields for four different linear accelerators. The method developed is easy to implement and can be used for regular linear accelerator quality assurance for VMAT.

## II. MATERIALS AND METHODS

### A. Materials

#### A.1 VMAT test plan

**Figure 1:**
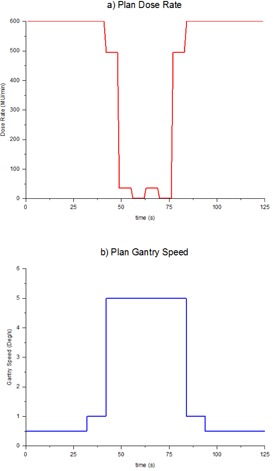
Dose rates (a) and gantry speeds (b) utilized in the VMAT test plan.

A Varian Trilogy and three 21iX linear accelerators (Varian Medical Systems) operating in the 6 MV photon mode were used for all irradiations. To perform measurements a VMAT test plan was required where the MLC had been retracted to allow an open field. This was achieved by modifying the Varian VMAT customer acceptance plan “RA CAP MIL 6X 600DR” using the MATLAB programming language and software (MathWorks Inc., Natick, MA). For this purpose the MATLAB program was simply used as a DICOM editor. Within the DICOM header of the plan the central MLC leaf positions were manually altered so that they were retracted beyond a 20 by 20 cm^2^ jaw‐defined field. The plan was run using DICOM RT mode. The VMAT CAP plan was chosen as it utilizes the extremes of allowed dose rate and gantry speeds and also incorporates zero dose sectors. The plan operates in the counterclockwise direction, but a version was also modified to run in the clockwise direction. The modified plans (see Fig. 1) incorporate the following features:

- Arc range: 128° to 232° gantry rotation (and vice versa for clockwise rotation)
- Dose rates: 600, 494, 35, and 0 MU/min
- Gantry speeds: 0.5, 1.0, and 5.0°/s

### B. Measurement Methods

To determine whether the beam symmetry varied during VMAT delivery, a method of measuring time resolved symmetry was required. To achieve this, a 2D array with cine mode acquisition and a stable gantry mount was required. Both the IBA MatriXX (IBA Dosimetry, Schwarzenbruck, Germany) and Sun Nuclear IC profiler (Sun Nuclear Corporation, Melbourne, FL) were investigated for this task. It was soon found that in cine mode the dynamic range of the MatriXX meant that prohibitively large sampling times were required to produce images with acceptably low noise for symmetry measurements.

The IC profiler is a 2D ion chamber array specifically designed for symmetry measurements. The IC profiler can be mounted to the collimator and utilizes ion chambers separated by 0.51 cm in both the in‐plane and cross‐plane directions, which allow measurements for up to 32 by $32\;{\text{cm}}^{2}$ field size at isocenter. The IC profiler has been previously characterized by Simon et al.^(9)^ In Simon et al's study, comparison was made between IC profiler profiles and those measured using an IBA CC13 ionization chamber in a water phantom. The error spread in profile comparison between the profiler and scanning water tank was found to be approximately ±0.75%.

To validate the IC profiler for time‐resolved symmetry during VMAT measurements, a series of measurements were performed with beams of increasing complexity until the modified VMAT CAP plan was measured. The first step in the process was to compare the symmetry measured from an IC profiler cine frame representative of the series at a point where the steering servos had corrected the beam with the symmetry derived from a gold‐standard water tank ion chamber scan. This was done using in‐plane and cross‐plane water tank scans with the following parameters: 6 MV, 600 MU/min, chamber effective point of measurement at $d_{\text{max}}$ (1.5 cm), 98.5 cm SSD, 20 by $20\;{\text{cm}}^{2}$ field size, CC13 chamber. Gantry and collimator=0

The IC profiler was irradiated in the gantry mount and solid water buildup was added to place the effective point of measurement of the detectors at $d_{\text{max}}$ (1.5 cm) and 98.5 cm SSD. The gain was set to the standard setting of 4 and the cine frame rate of the system is 8 frames/s. For analysis purposes every 8 frames were averaged to provide 1 measurement/s. These parameters were found to optimize the balance of temporal resolution and signal‐to‐noise and were used for all subsequent IC profiler measurements. The symmetry was determined by extracting the raw profiles into MATLAB and using a script that included interpolation of the ion chamber readings to provide 0.1 mm resolution. For the purposes of this study, symmetry is calculated from the measured ratios of all points along the profile symmetric about central axis and within the flattened area of the beam. These ratios always use the left side as the numerator and are multiplied by 100 so that perfect symmetry is denoted as 100%. The reported symmetry is the measurement with the greatest variation from 100%.

After comparing the IC profiler cine frame symmetry to the symmetry measured in the water tank, the rest of the measurements build on each other in terms of linac variables towards VMAT. All measurements were performed across the four different linacs.

The first step is to use the IC profiler to measure time‐resolved symmetry throughout a static gantry exposure with 600 MU/min at the cardinal gantry angles. At this point slop in the gantry mount could possibly distort the measurements. In this study the center of the profiles is calculated from the 50% points on the penumbra. Therefore any slop in the gantry mount in either the lateral or longitudinal directions of the device won't have any effect on the measured symmetry. Tilting of the array is a possible cause of erroneous symmetry and this was checked by placing a spirit level on the array surface at the cardinal gantry angles. No apparent difference was detected on the spirit level at the cardinal gantry angles.

The next step is for the measurement of time‐resolved symmetry during a conformal arc in both clockwise and counterclockwise gantry rotation. This measurement adds rotating gantry and the associated variability of gravitational forces on the linac components during the beam, but does so at constant gantry speed and constant dose rate of 600 MU/min.

The last measurement in the series is for time‐resolved symmetry measurement during the modified VMAT CAP plan where there is both dose‐rate and gantry‐speed modulation. Measurements were performed for both clockwise and counterclockwise gantry rotation. Short‐term reproducibility was tested by taking three consecutive measurements and comparing.

## III. RESULTS

### A. IC profiler compared to CC13 ionization chamber

The symmetry measured by the IC profiler during a representative cine frame was compared to the gold‐standard CC13 ionization chamber scan in the water tank (see Table 1).

**Table 1:** Symmetry measured with IC profiler cine mode and with CC13 ion chamber in the water tank

	*IC Profiler Symmetry (%)*	*Ion Chamber Symmetry (%)*	*% Difference*
cross‐plane	100.8	101.1	0.3
in‐plane	100.7	100.8	0.1

### B. Time‐resolved symmetry during static gantry fields

**Figure 2:**
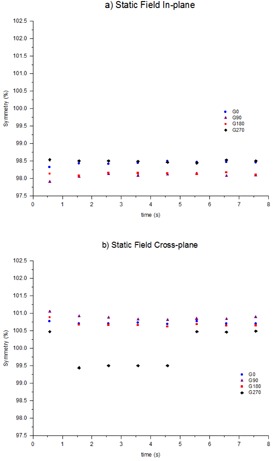
Time‐resolved symmetry during static gantry, open fields at the four cardinal gantry angles: (a) in‐plane, (b) cross‐plane. Results have been presented for one linac for publishing purposes, but are indicative of all four linacs.

The time‐resolved symmetry during beam on was measured for static gantry open fields at the cardinal gantry angles. Figure 2 shows that in general the beam symmetry stays constant throughout the exposure to within 0.3%. The exception is the cross‐plane result at gantry 270° in Fig. 2(b). For this exposure the symmetry starts at approximately 100.5% before making a sudden jump to 99.5% then returning to 100.5% towards the end of the exposure. This effect was reproducible and similar effects were also seen on other linacs.

Figure 2 also demonstrates that the symmetry between the cardinal gantry angles is consistent to within 0.7% with the exception again of gantry 270° in the cross‐plane direction. When averaged, the in‐plane symmetry at gantry 0° is 98.3%. This is in agreement with a CC13 ion chamber measurement taken in the same week, which also measured symmetry at 98.3%. For cross‐plane, the IC profiler measured asymmetry at 100.7% while the CC13 measured symmetry was 101.1%. This level of agreement is consistent with the results presented in Table 1.

### C. Time‐resolved symmetry during conformal arcs

**Figure 3:**
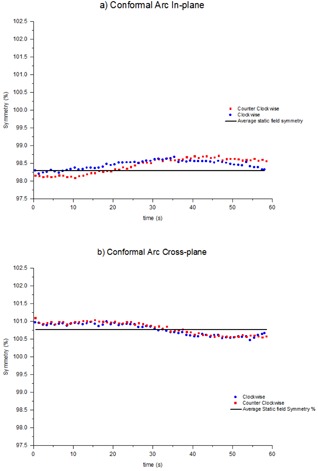
Time‐resolved symmetry during clockwise and counterclockwise conformal arcs, (a) in‐plane, (b) cross‐plane. Results have been presented for one linac for publishing purposes, but are indicative of all four linacs.

The time‐resolved symmetry was measured during a conformal arc which included constant speed gantry motion and constant dose rate. Figure 3 shows how the symmetry changed during clockwise and counterclockwise conformal arcs in the in‐plane and cross‐plane directions. The clockwise results have been reversed so that the clockwise and counterclockwise results correlate with the same gantry angles. The in‐plane and cross‐plane results agree to within 0.3% and, in both planes, there is a drift in symmetry during the arc of approximately 0.5%.

### D. Time‐resolved symmetry during a VMAT test plan

**Figure 4:**
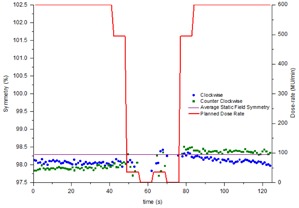
Time‐resolved in‐plane symmetry during a VMAT test plan.

**Figure 5:**
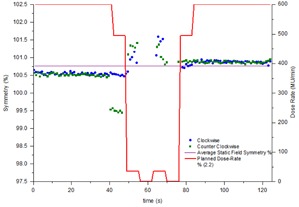
Time‐resolved cross‐plane symmetry during a VMAT test plan. Results have been presented for one linac for publishing purposes, but are indicative of all four linacs.

**Figure 6:**
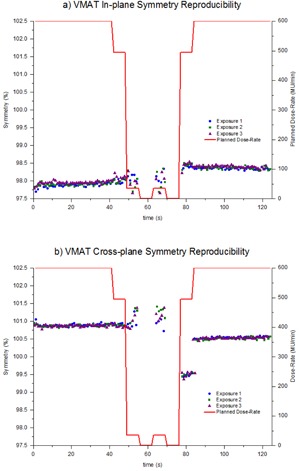
Time‐resolved symmetry short‐term reproducibility during a VMAT test plan: (a) in‐plane, (b) cross‐plane.

**Figure 7:**
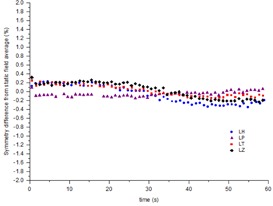
Time‐resolved in‐plane symmetry for a clockwise‐rotating conformal arc across four linacs.

**Figure 8:**
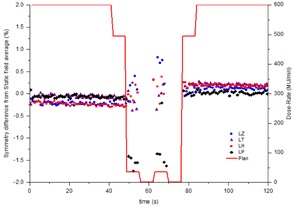
Time‐resolved in‐plane symmetry for a clockwise‐rotating VMAT test plan across four linacs.

**Figure 9:**
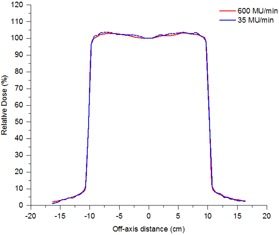
In‐plane profiles from the clockwise‐rotating VMAT test plan for linac LP from individual frames taken at the 20‐s mark in the 600 MU/min region and at the 52‐s mark in the 35 MU/min region.

The time‐resolved symmetry was measured during a modified VMAT test plan that includes both gantry speed and dose rate modulation. In Figs. 4 and 5 the symmetry measurements are plotted alongside the VMAT plan in terms of dose rate so that any change in symmetry with change in dose rate can be observed. The VMAT plan incorporates two zero‐dose‐rate regions, which represent avoidance sectors within the plan. Within such regions, symmetry is obviously meaningless, so results have not been included. Figure 6 indicates that the VMAT symmetry measurements are reproducible across three successive measurements to within 0.3% for the maximum dose rate regions and within 0.5% in the low dose rate and hence low signal‐to‐noise regions. Figure 7 demonstrates how consistently between linacs the continuously rotating gantry affects the beam symmetry. The results have been normalized to the static gantry field symmetry for each linac to take out the effect of variations in beam steering between the linacs for ease of viewing. Results have been presented for cross‐plane symmetry and a clockwise rotating gantry, but are also representative of both in‐plane and both directions of gantry rotation. Figure 7 indicates that the effect on symmetry of rotating the gantry is consistent between the four linacs to within ±0.4%. Figure 8 shows how beam symmetry changes during a VMAT test plan with dose rate and gantry speed modulation across four different linacs. Figure 8 shows that in the high dose rate regions the symmetry stays within ±0.5% for all four linacs, but there is greater variation in the low dose rate regions. Linac LP changed in symmetry by up to 1.75% when the dose rate drops to 35 MU/min and linac LZ changed by about 0.75% in the same region. Figure 9 shows profiles taken in the in‐plane direction during the clockwise‐rotating VMAT test plan on linac LP. One profile is from a frame taken in the sTable 600 MU/ min region, while the other is representative of a profile from a frame during the 35 MU/min region where a greater variation in measured symmetry is observed. The 600 MU/min profile indicates symmetry of −0.02% different from the static field average symmetry and the 35 MU/ min profile indicates symmetry difference of −1.75%. The 35 MU/min profile is visibly more “lumpy” than the 600 MU/min profile, which indicates reduced signal‐to‐noise ratio in the low dose rate regions. This is likely a potential contributor to the measured changes in symmetry measured in the low dose rate regions.

## IV. DISCUSSION

The comparison between symmetry measured using a CC13 ionization chamber in a scanning water tank and the symmetry measured from IC profiler profiles for a single cine frame agree to within 0.3%. With this level of agreement the IC profiler was deemed acceptable for the requirements of this study. When the IC profiler was used to measure time resolved symmetry during a static gantry field it was found that the symmetry was generally constant during beam‐on to within 0.3% and comparison between measurements at the four cardinal gantry angles indicates agreement in symmetry to within 0.7%. This discrepancy is likely caused by a combination of small variations in the measurement due to the sag in the gantry mount, plus real variations due to sag of linac components such as steering coils under the different gravity conditions present at the different gantry angles. The exceptional measurement presented in Fig. 2(b) is the cross‐plane $\text{gantry}=270^{\circ }$ measurement. Within this exposure the symmetry starts at 100.5% before changing to 99.5% and then returning to 100.5% towards the end of the exposure. The effect was found to be reproducible and similar results were recorded on other linacs (not presented). No good explanation is given for the sudden change in symmetry. It is possible that it is a result of the beam steering servo system's operation. It should be remembered that 0.5% asymmetry is quite small with regards to symmetry and, anecdotally, it is difficult to steer the beam to better than 0.5% symmetry due to limitations on the resolution of the linac potentiometers. Also, since the symmetry is based upon equidistant measurement points from both sides of the profile than if the angle servo steers the beam to change symmetry by 0.5%, then this equates to only a 0.25% change in either side of the profile compared to central axis.

The symmetry during clockwise and counterclockwise conformal arcs was found to agree within 0.3%. During the conformal arcs, the symmetry was found to vary by up to 0.5% (Fig. 3). Clockwise and counterclockwise results were in agreement to within 0.3%, indicating that the variation during the exposure is related to gantry angle. This is in agreement to the gantry angle dependency indicated in Fig. 2 for static gantry fields at the cardinal gantry angles. At about 30 s in the exposure (approximately gantry 0°), the in‐plane and cross‐plane asymmetry is measured to be approximately 98.6% and 100.8%, respectively. These results are both within 0.3% of the CC13 measured symmetry value.

The symmetry variation during the VMAT test plan with variable dose rate and gantry speed results of Figs. 4 and 5 shows that, within the maximum dose rate regions (600 MU/min), the symmetry results appear in agreement with the conformal arc results. The symmetry values are consistent and the drift in symmetry with gantry angle is also apparent. For the in‐plane results of Fig. 4, within the modulated dose rate regions the symmetry values still appear to follow the trend from the conformal arcs, although there is greater variation between adjacent measurement points. This is to be expected considering that there is reduced signal‐to‐noise in the low dose rate regions. In the cross‐plane direction, the trend from the conformal arcs isn't followed in the dose rate modulated sections. In these regions asymmetry is increased up to 1.0%. In Fig. 5(b) within the second 494 MU/min region, the symmetry is 99.5% and is not consistent with the conformal arc results. However, at the point where the dose rate in the plan increases to 600 MU/min, the symmetry changes to 100.5%, consistent with the conformal arc results. This jump in symmetry is similar to the jump evident in Fig. 2(b) for the static gantry cross‐plane measurement at $\text{gantry}=270^{\circ }$. Since in the VMAT plan the jump coincided with a change in dose rate, then this phenomena may be related to the dose rate servo. Figure 6 demonstrates that, in the short term, this jump is reproducible. The results of Fig. 6 indicate that, in the short term, the symmetry during the VMAT test plan is reproducible to within 0.3% for the high dose rate regions and within 0.5% for the low dose rate regions.

The results presented in Figs. 7 and 8 indicate that, once variations in beam steering between different linacs is taken out, the effect on symmetry of variable rotating gantry and dose rate modulation are consistent across the four linacs measured. The variability is generally within 0.5% across all four linacs across the whole exposure for both a conformal arc with constant gantry speed and dose rate and for a VMAT test plan with gantry speed and dose rate modulation. The exception is within the low dose rate regions (35 MU/min) where the variation in symmetry is shown to increase by up to 1.75%. From the appearance of the profiles in Fig. 9, at least a component of this variation is likely due to low signal‐to‐noise ratio on the IC profiler, but the total effect is within the 2% tolerance suggested by NCS Report 24.^(8)^ Since the measured symmetry deviations are within the NCS tolerance and are likely to be primarily due to signal‐to‐noise issues in the measurement at low dose rates, it is expected that the dosimetric effect on patient delivery is likely to be negligible. If real, the effect would be most significant for plans with extremely high modulation, resulting in a high percentage of the plan being delivered with low dose rates.

## V. CONCLUSIONS

The IC profiler has been used to measure time‐resolved symmetry for a series of exposures of increasing complexity ranging from a simple static gantry open field, through a conformal arc with constant gantry speed and dose rate, to a VMAT test plan with variable gantry speed and dose rate. Symmetry was always measured to be within the 2% tolerance of NCS Report 24,^(8)^ and this study provides results and a practical test to meet the requirements of this report. The results indicate that beam symmetry during VMAT treatment delivery is maintained to clinically acceptable levels on Varian linacs. The test has been implemented into the departmental linac annual quality assurance program.

## ACKNOWLEDGMENTS

Special thanks to Dr Pejman Rowshanfarzad for providing the MATLAB script used to calculate the symmetry from the IC Profiler profiles.

## COPYRIGHT

This work is licensed under a Creative Commons Attribution 4.0 International License.
